# Genome Sequence of Three Siphoviruses in the EE, GA and EA5 Actinobacteriophage Clusters: Biscayne, Bush and GreenIvy.

**DOI:** 10.17912/micropub.biology.001397

**Published:** 2025-01-06

**Authors:** Xavier S Purroy, Betty R Sierra, Lara Becerra Reymundo, Victoria M Serradet, Alejandra M Camacho, Nicole A Briceno, Katherine Artiles, Pooja Lad, Nhan Phan, Alison Rodriguez Leiva, Jazlyn N Appolon, Akram Mikhail, Arianna M Ruiz, Carlos Rodriguez, David Vega, Gabriela Moyano, Grace Intrator, Kiryl Yasinski, Kristen Mclean, Nicole Gonzalez Giliberti, Erika Ramirez Ramirez, Victor Adolpho de Melo, Alexandra S Alsina, Maria Y Andino, Brian A Becker, Hillary Castellanos, Natalia A Castillo, Brandon S Fernandez, Jeremiah R Estinvil, Amanda A Gonzalez, Emily M Hernandez, Ayden Ho, Sheikh F Islam, Anna Liubenco, Lance Mejia, Sandra N Meesala, William Morales-Ramirez, Nathalie Morlote, Kevin Ramos-Homs, Jorge A Rodriguez, Leydis M Torres, Patricia Waikel, Jaime Mayoral

**Affiliations:** 1 Biological Sciences, Florida International University, Miami, FL. USA

## Abstract

Bacteriophages Biscayne, Bush and GreenIvy were isolated from soil samples in Miami, FL using *Microbacterium foliorum* NRRL B-24224 as host. Transmission electron microscopy shows siphoviral morphologies for all three phages. Based on gene content similarity to other actinobacteriophages, they are assigned to the EE, GA and EA5 clusters, respectively.

**Figure 1. Transmission electron microscopy and plaques of Biscayne, Bush and GreenIvy:**
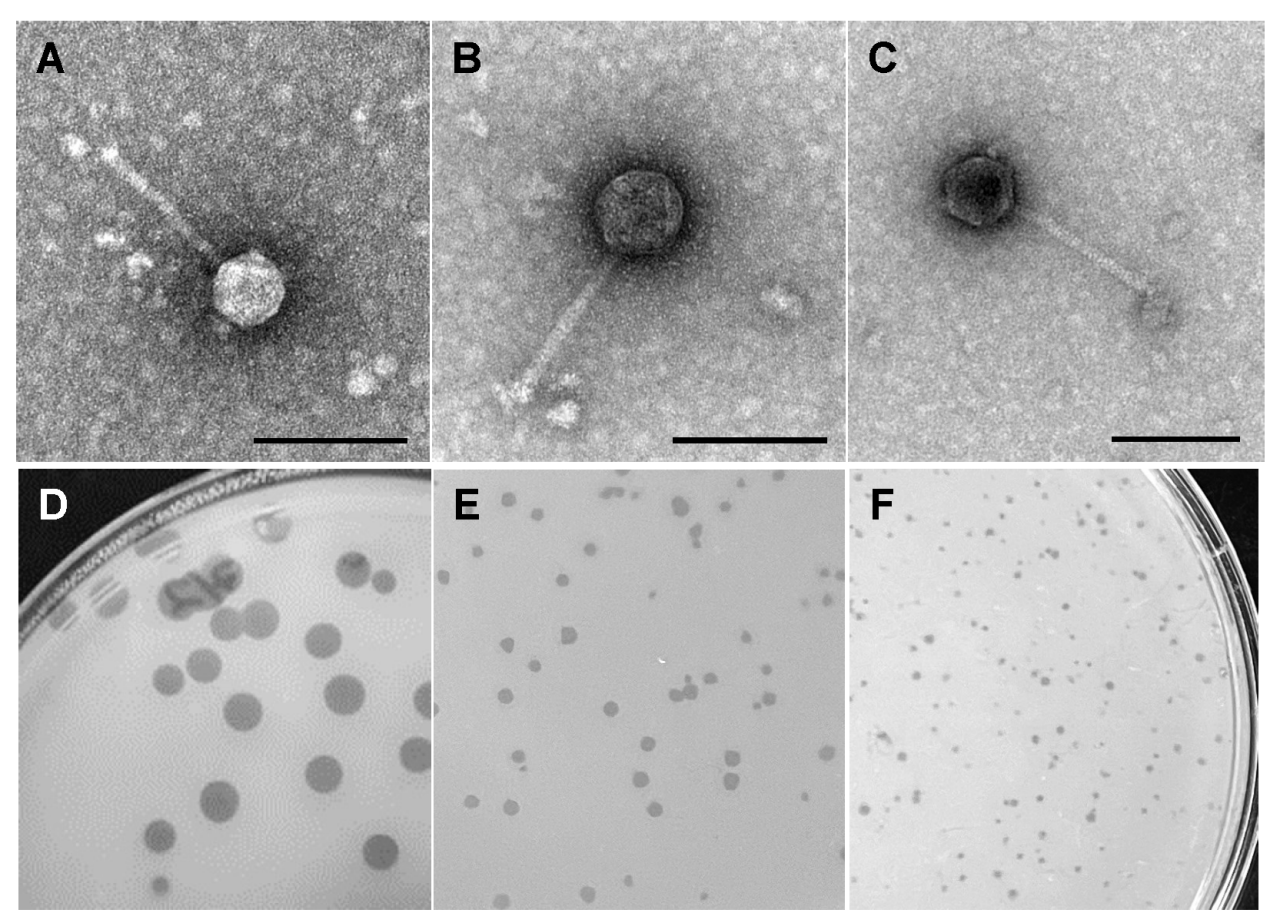
Transmission electron microscopy of (A) Biscayne, (B) Bush and (C) GreenIvy revealed siphovirus morphologies. Scale bars = 100 nm. Plaque assays show all three phages to form clear plaques of different sizes (D) Biscayne, (E) Bush and (F) GreenIvy. Electron Microscopy micrographs were taken using a Hitachi HT7800 120 kV TEM with an AMT Nanosprint15 B digital camera.

## Description

*Microbacterium foliorum* is an alkali-tolerant, gram-positive, and aerobic bacterium that was originally isolated from the phyllosphere of grasses (Kim et al., 2018) . Its fast growth rate and simple nutrient requirements make it an ideal host for bacteriophage discovery under laboratory conditions to enable a deeper exploration of the vast diversity of bacteriophages (Russell et al., 2019) .

Phages were isolated from soil collected from different areas of Miami, Fl. Biscayne was isolated from near a pond in Florida International University's Biscayne Bay Campus, Bush from the city of Doral and GreenIvy from Florida International University's Modesto Maidique Campus (GPS coordinates, **Table 1** ). Soil samples were suspended in peptone-yeast extract-calcium (PYCa) liquid medium, centrifuged at 2,000 *g* and the supernatant was filtered through a 0.22 μm filter. Filtrates were incubated with *M. foliorum* NRRL B-24224 and incubated at 30°C for 48 hours. Enriched samples were plated in PYCa top agar with *M. foliorum* and individual plaques were selected and purified through two rounds of plating. Plaque and particle characteristics are provided in Table 1. Transmission electron microscopy of lysates showed a siphovirus morphology for all three phages isolated ( **Fig. 1** ).

Phage particles in lysates were concentrated by precipitation with ZnCl _2_ before DNA was extracted using the Wizard ^®^ Genomic DNA purification kit (Promega). DNA was prepared for sequencing using the NEB Ultra II Library kit and sequenced using Illumina Sequencing (v3 reagent), generating 150-base single ends reads that were assembled using Newbler v2.9 and genomes checked for completeness using Consed (Gordon et al., 1998; Russell & Hatfull 2017) . Sequencing data and genome characteristics for each phage are described in **Table 1** .

Genomes were annotated using DNA Master v5.23.6 Build 2705 (cobamide2.bio.pitt.edu). Glimmer v3.2 (Delcher et al., 2007) , Genemark v3.25 (Besemer & Borodovsky 2005) , Starterator v1.2 (http://phages.wustl.edu/starterator), BLASTp (Altschul et al., 1990) using the Actinobacteriophage and NCBI non-redundant database, HHpred (Söding et al., 2005) using the PDB_mmCIF70, Pfam- v.36, NCBI Conserved Domains databases, Phamerator (Cresawn et al., 2011) , and TMHMM v2.0 (Krogh et al., 2001) were used to annotate putative gene starts, functions and transmembrane domains. Aragorn v2.41 (Laslett & Canback 2004) and tRNAscanSE v2.0 (Lowe & Eddy 1997) were used to identify tRNAs. Phages were assigned to clusters based on gene content similarity of at least 35% to phages in the Actinobacteriophage database, phagesdb (https://phagesDB.org) (Pope et al., 2017; Russell & Hatfull 2017) . All software were used with default parameters.

Biscayne's 17,529 bp long genome contained 25 putative protein-encoding genes, from which 19 (76%) were assigned a putative function. Biscayne is assigned to phage cluster EE, that includes phages with small genomes ranging from 16-18 kbp. Consistent with phages in this cluster, the majority of Biscayne's genome encodes structure, assembly and lysis functions, including a fusion of the major capsid protein and the capsid maturation protease. Among the remaining genes are 4 that encode for helix-turn-helix proteins. A -1 translational frameshift was identified at bp 7,339 with a putative slippery sequence GGGAAA. Biscayne's genome length was the second largest of the 131 members in the EE cluster.

Bush, on the other hand, is one of only 9 phages in cluster GA, to date. It has a 38,879 bp long genome containing 67 predicted protein coding genes, of which 30 (45%) were assigned a function. Two genes in Bush, gene 35 (441 bp) and gene 65 (414 bp), have no homologues in the Actinobacteriophage database. As with other GA phages, all genes in the genome are transcribed in the same direction, with the exception of a single tRNA (Gly), and encode two tail assembly chaperones from a single gene using a programmed translational shift. In Bush, it is a -1 frameshift at bp 11,135 bp within the GGGAAAA slippery sequence.

As is typical of subcluster EA5 phages, GreenIvy encodes two tail assembly chaperones from a single gene using a -1 programmed translational shift (at bp 9,420) and a single tRNA (Ala, TGC), with virion structure, assembly and lysis genes transcribed in one direction (-->), and DNA metabolism genes, along with many genes of unknown function, transcribed in the opposite direction (<--). The genome is 40,254 bp long and contains 61 genes (33 reverse and 28 forward). Twenty-four genes (39%) were assigned a function.

No immunity repressor or integrase functions could be identified across all three genomes, suggesting the phages presented in this study are unlikely to establish lysogeny.

**Nucleotide sequence accession numbers**

Biscayne is available at GenBank Accession No. is PP978855 and Sequence Read Archive (SRA) No. SRX24338403 . Bush is available at GenBank Accession No. is PP978824 and Sequence Read Archive (SRA) No. SRX24338413 . GreenIvy is available at GenBank Accession No. is PP978818 and Sequence Read Archive (SRA) No SRX24338415 .

**Table 1.**

**Table:** 

**Phage**	**Biscayne**	**Bush**	**GreenIvy**
Phage Collection Coordinates	25.90988 N, 80.13763 W	25.81692 N, 80.37672 W	25.754449 N, 80.374755 W
Plaque Size (mm) (N=5)	5	2.5	1
Capsid Diameter (nm) (N=3)	50	60	54
Tail Length (nm) (N=3)	100	105	135
Number of 150-base sequencing reads	293,794	311,820	291,356
Shotgun Sequencing Coverage	2,400	1,139	1,026
Genome Size (bp)	17,529	38,879	40,254
GC Content (%)	68.7	68.0	64.0
Genome End Type	3' single-stranded overhang: 5'-CCCGCCCCA	circularly permuted	circularly permuted
Cluster	EE	GA	EA5
Number of Putative Protein Coding Genes	25	67	61
Number of Genes Assigned a Putative Function	19	30	24
Number of tRNAs	0	1	1
